# A cross-sectional study on psychological impact of acne vulgaris

**DOI:** 10.6026/973206300221473

**Published:** 2026-03-31

**Authors:** Devesh Kumar Vyas, Sanjay Prasad, Preetam Singh Katroliya, Vishnu Kumar Gupta, Parul Nema

**Affiliations:** 1 Department of Psychiatry, SRVS Medical College, Shivpuri, Madhya Pradesh, India; 2 Department of Psychiatry, Bundelkhand Medical College, Sagar, Madhya Pradesh, India; 3 Department of Dermatology, SRVS Medical College, Shivpuri, Madhya Pradesh, India; 4 Department of Community Medicine, SRVS Medical College, Shivpuri, Madhya Pradesh, India; 5 Department of Pathology, SRVS Medical College, Shivpuri, Madhya Pradesh, India

**Keywords:** Acne vulgaris, depression, anxiety, quality of life, psychological impact, acne severity

## Abstract

Acne vulgaris is a common dermatological condition that often affects psychological well-being, with depression, anxiety and reduced quality of life being frequently observed. Therefore, it is of interest to evaluate the psychological impact of acne and its relationship with clinical severity in the Indian population. A cross-sectional study was conducted among 207 patients, assessing psychological status using established tools. Results showed a positive correlation between acne severity and higher levels of depression, anxiety and poorer quality of life. This research advances knowledge by highlighting the need for integrated care that addresses both the dermatological and psychological aspects of acne.

## Background:

Acne vulgaris is a chronic inflammatory disorder of the pilosebaceous unit that affects the majority of adolescents and a substantial proportion of adults worldwide, often extending into early adulthood and beyond [1]. Although traditionally considered a cosmetic or benign dermatological condition, an extensive body of evidence demonstrates that acne can significantly affect psychological well-being, self-esteem and quality of life [1, 2]. Patients with acne frequently report symptoms of anxiety and depression that exceed those observed in unaffected individuals, contributing to social withdrawal, negative body image and emotional distress [2, 3]. Quality of life impairment in acne is not limited to emotional domains but also includes disruptions in daily functioning, social interactions and self-confidence. Studies using validated instruments such as the Dermatology Life Quality Index (DLQI) have shown that higher scores reflecting worse dermatologic quality of life are often accompanied by increased anxiety and depressive symptoms [1, 2]. The psychosocial burden is multifaceted and may be influenced by factors such as lesion visibility, scar formation and perceived stigma, which collectively exacerbate mental health sequelae [3]. Despite this recognized psychological impact, the relationship between the clinical severity of acne and psychological outcomes remains heterogeneous across studies. While some investigations report a positive association between greater acne severity and worse psychological measures, others have found only modest or inconsistent correlations, suggesting that individual characteristics and psychosocial context may modulate this relationship [4, 5]. Therefore, it is of interest to quantify the psychological impact of acne vulgaris and examine its association with clinical severity using validated assessment tools.

## Methodology:

This cross-sectional observational study was conducted at a tertiary care hospital in India, adhering to the principles outlined in the Declaration of Helsinki. Written informed consent was obtained from all participants prior to enrollment. Patients aged 16 to 35 years with clinically diagnosed acne vulgaris, both male and female, were considered for inclusion, while those with chronic dermatological conditions, systemic illnesses affecting the skin, ongoing psychiatric treatment, or a history of major trauma or surgery in the past three months were excluded. Pregnant and lactating women were also excluded to avoid confounding psychological parameters. A pilot study suggested a prevalence of moderate to severe psychological distress in approximately 40% of acne patients and using this proportion, a 95% confidence interval and a margin of error of 7%, the minimum required sample size was calculated to be 190 participants. Accounting for potential non-response, 220 patients were approached, of whom 207 completed the study and were included in the final analysis. Demographic data were recorded using a structured pro forma and acne severity was assessed by a senior dermatologist using the Global Acne Grading System (GAGS). Psychological status was assessed using validated scales, including the Beck Depression Inventory-II (BDI-II), the State-Trait Anxiety Inventory (STAI) and the Dermatology Life Quality Index (DLQI), with all questionnaires administered in the local language. Data were analyzed using SPSS version 26, with associations between acne severity and psychological parameters analyzed using Pearson's correlation coefficient. Differences in psychological scores between genders and severity groups were assessed using independent t-tests or one-way ANOVA, with a p-value <0.05 considered statistically significant.

## Results:

A total of 207 participants completed the study. The cohort had a mean age of 22.6 years, with a slightly higher proportion of males. Most participants were students and had attained at least a graduate-level education. Mild-to-moderate acne was the most frequently observed clinical severity (Table 1 - see PDF). Assessment of psychological parameters indicated that participants experienced mild-to- moderate depressive and anxiety symptoms, along with a moderate impairment in dermatology-specific quality of life (Table 2 - see PDF). Analysis of the relationship between acne severity and psychological outcomes revealed statistically significant positive correlations for all measures, suggesting that higher clinical severity is associated with greater depressive symptoms, elevated anxiety levels and worse quality of life (Table 3 - see PDF). This association is further illustrated in Figure 1 (see PDF), which demonstrates a progressive increase in mean psychological scores with increasing acne severity. Comparison of psychological scores between male and female participants did not show statistically significant differences across depression, anxiety or quality of life measures, indicating that the observed psychological impact was largely independent of gender (Table 4 - see PDF). Collectively, these findings highlight a clear relationship between the severity of acne and the magnitude of psychological burden, underscoring the importance of addressing mental health aspects in the management of acne vulgaris.

## Discussion:

In this study, acne vulgaris was associated with notable psychological burden, particularly in terms of depressive symptoms, anxiety and reduced dermatology-specific quality of life. The significant positive correlations observed between acne severity and all psychological parameters align with evidence from recent observational studies indicating that greater clinical severity of acne is linked with an increased risk of depression and anxiety in patients with acne [6, 7]. For instance, a population-based survey involving patients with varying grades of acne demonstrated that individuals with more severe presentations reported higher anxiety and depressive symptom scores and worse well-being compared with those with mild acne [8]. The relationship between acne severity and psychological outcomes is further supported by data showing that greater stigmatization in acne correlates with both increased depressive and anxiety symptoms and reduced quality of life, irrespective of patient age [9]. These findings highlight that acne's influence extends beyond physical symptoms, contributing significantly to psychosocial stressors that can perpetuate negative emotional states. Such psychosocial impact is consistent with previous cross-sectional research demonstrating that impaired quality of life in acne patients often accompanies heightened psychological distress [10]. Although some studies have reported inconsistent associations between acne severity and psychological distress-suggesting that factors such as coping capacity, cultural context and assessment tools might modulate these relationships [11] the weight of current evidence supports a trend wherein more severe acne corresponds with greater mental health burden. Our gender comparison, which did not show statistically significant differences, further underscores that the psychological impact of acne transcends sex, although other cohorts have observed gender linked variations in anxiety and depression, particularly in specific cultural or demographic contexts [12]. Collectively, these observations emphasize the need for clinicians to adopt a holistic approach to acne management that incorporates routine psychological evaluation and, when indicated, appropriate referral for mental health support. Addressing psychosocial dimensions alongside dermatological therapy may improve overall patient outcomes and quality of life.

## Conclusion:

Acne vulgaris is associated with a substantial psychological burden, with increasing clinical severity correlating with higher levels of depression, anxiety and impaired quality of life. Thus, we show the importance of holistic management strategies that address both dermatological and mental health aspects, highlighting the need for routine psychological screening and supportive interventions in patients with moderate to severe acne.
